# Macroscopic Hematuria—A Leading Urological Problem in Patients on Anticoagulant Therapy: Is the Common Diagnostic Standard Still Advisable?

**DOI:** 10.5402/2012/710734

**Published:** 2012-04-01

**Authors:** Artur A. Antoniewicz, Łukasz Zapała, Sławomir Poletajew, Andrzej Borówka

**Affiliations:** Department of Urology, Medical Centre of Postgraduate Education, Miedzylesie Hospital, 04-749 Warsaw, Poland

## Abstract

All urological standards of care are based on the past definition of the clinical importance of macroscopic hematuria. The aim of the study was to assess the phenomenon of iatrogenic hematuria in current clinical practice and analyze its origins in patients receiving anticoagulant drugs. Retrospective analysis of clinical documentation of 238 patients that were consulted for hematuria in 2007–2009 by 5 consultant urologists was performed. In the group of 238 patients with hematuria, 155 (65%) received anticoagulants. Abnormalities of urinary tract were found in 45 (19%) patients. Estimated cost of a single neoplasm detection reached the value of 3252 Euro (mean 3-day hospitalization). The strong correlation between the presence of hematuria and anticoagulant treatment was observed. Authors suggest to redefine the present and future role of hematuria from a standard manifestation of serious urological disease to a common result of a long-term anticoagulant therapy.

## 1. Introduction

Hematuria, defined as a clearly visible change in urine color due to blood additives, may be a symptom of serious urinary tract disease. Thus, it should always be an urgent diagnostic matter for a clinician. The usage of anticoagulant or antiplatelet drugs is beneficial for patients with several diseases. However, serious complications may appear during such a therapy, including mucosal bleeding in the form of hematuria. Iatrogenic hematuria may be the reason for urological consultation and hospitalization in urological department, during which standard diagnostic procedures are usually performed.

All urological standards of care are based on the past definition of the clinical importance of presence of micro- and macroscopic hematuria. The growing number of iatrogenic hematuria reported in literature requires verification of guidelines for the management of patients with hematuria receiving any anticoagulant therapy. The aim of the study was to assess the phenomenon of iatrogenic hematuria in current clinical practice and analyze its origins in patients receiving anticoagulant drugs. The economic aspects of diagnosing hematuria were of special interest.

## 2. Material and Methods

**Figure 1:**
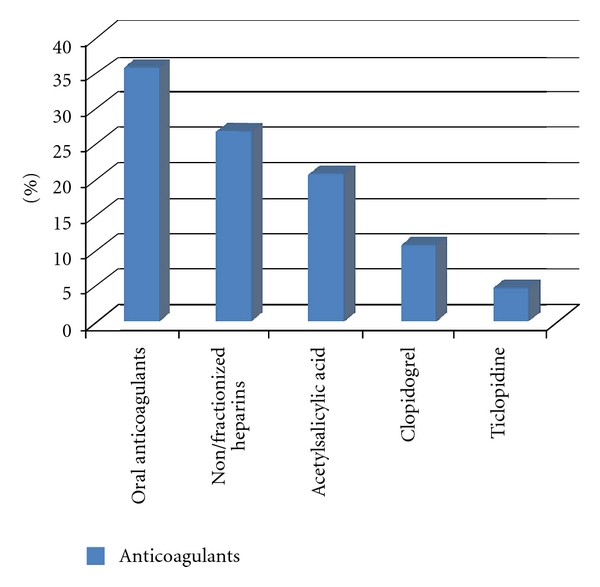
Individuals that received anticoagulant drugs—group A.

Retrospective analysis of clinical documentation of 238 patients (132 men, 106 women) aged 18–99 years (average 57 years) was performed. All patients were consulted for hematuria in 2007–2009 by 5 consultant urologists in the following departments: Nephrology (43), Cardiology (27), General (23) and Vascular Surgery (11), Cardiosurgery (40), Hematology (34), Gynecology (11), and Internal Diseases (49). In the group of 238 patients with hematuria, there was a division on the subgroup of 155 (65%) individuals, who received anticoagulant drugs—group A (Figure 1): oral anticoagulants (36%), nonfractionized or fractionized heparins (27%), and antiplatelet drugs: acetylsalicylic acid (21%), clopidogrel (11%), and ticlopidine (5%). Another 83 (35%) subgrouped patients, who presented with hematuria—group B, did not receive any anticoagulant drugs. Hematuria was found predominantly in patients over 65 years (67%), with concomitant diseases (71%): hypertension, coronary heart disease, arrhythmia, and end-stage renal disease. The majority of patients had the full diagnostic panel performed (*n* = 216, 91%) including USG of urinary tracts, urography or contrast CT, and cystoscopy. The diagnostic results were analyzed with the special attention to the negative ones, in which no abnormalities in urinary tracts were found (*n* = 209, 88%). Statistical analysis was performed using chi-square test with Yates correction.

## 3. Results

**Figure 2:**
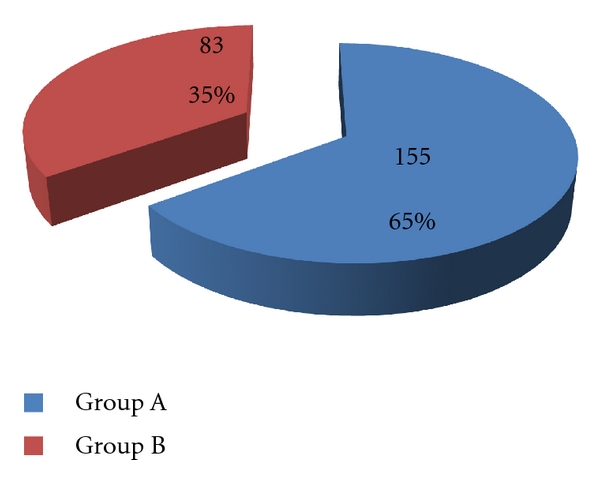
Hematuria as the top reason for urological consultation: the existence in group A versus group B.

**Figure 3:**
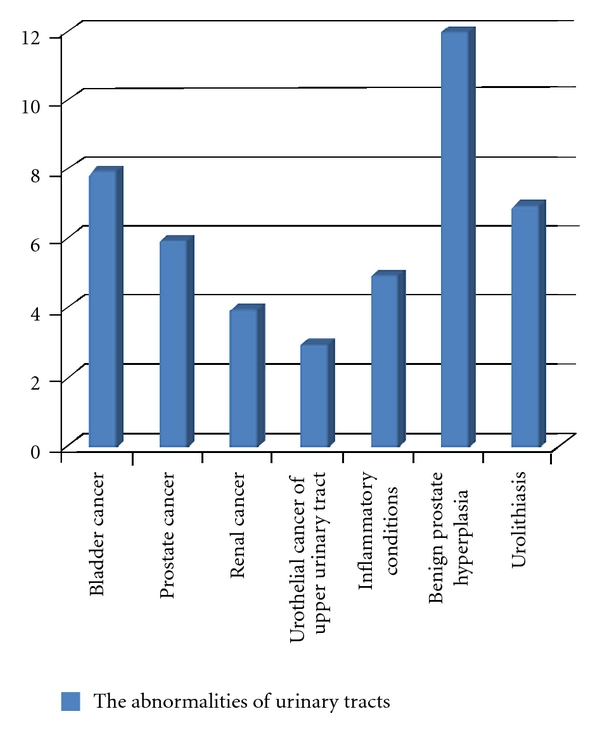
Abnormalities of urinary tract that were found in overall (group A plus group B).

Hematuria was the top reason for urological consultation (238/871, 27%). It occurred predominantly in individuals on anticoagulant drugs representing group A (65% versus 35%) (Figure 2). The diagnostic panel of greatest clinical importance was ultrasound examination and cystoscopy. Abnormalities of urinary tract were found in overall (group A plus group B) in 45 (19%) patients (Figure 3): neoplasms—bladder cancer (8), prostate cancer (6), renal cancer (4), and urothelial cancer of upper urinary tract (3); inflammatory conditions (5); benign prostate hyperplasia (12); urolithiasis (7). The number of pathologies detected in the group A compared with group B was 8% and 16%, respectively (*P* = 0.2). The common probability for A + B groups of finding a tumor was low (*P*1 = 0.1 in men, *P*2 = 0.06 in women) with the highest one for diagnosing bladder cancer (*P*3 = 0.037 in men, *P*4 = 0.02 in women). The estimated cost of diagnostic procedures for hematuria per patient was 287 Euro, assuming average time of diagnostics as a 3-day hospitalization (1EUR = 4.5 PLN). The cost-effectiveness analysis (number of patients × cost of a single diagnostic panel/number of neoplasms detected) revealed that the cost of a single neoplasm detection reaches the value of 3252 Euro, which remains unacceptable for any health provider worldwide taking into consideration a predominant number of cases of iatrogenic hematuria (Table 1). 

## 4. Discussion

Anticoagulants are increasingly used for the prevention and treatment of thromboembolic complications of vascular diseases [1]. Bleeding from the urinary tracts is naturally one of the most important complications of such a therapy.

Hematuria occurred to be the main reason for consultations in our material, being the matter of urological visits in various departments in 27% of cases. Hematuria is claimed to involve 4% to 20% of all urological visits [2]. Those findings differ slightly, but our patients made up a specific group of great anticoagulants uptake due to serious health conditions (cardiology, cardiosurgery, and vascular surgery). Hematuria in a review of observational studies, average annual rates of fatal, and major and major/minor bleeding was 0,8, 4,9, and 15%, respectively [3]. Gross hematuria occurred in 2,5% of the population [4]. However, the rate was higher in the older patients and when the indication for anticoagulant treatment was arterial disease [5]. About one-third of patients, who had bleeding complications, had more than one indication for anticoagulants, for example, peripheral and/or cerebral arterial disease, ischaemic heart disease, atrial fibrillation, and venous thromboembolic disease [5]. It is consistent with the results obtained in our study, in which in the majority of cases patients over 65 years were consulted, while the main reason for anticoagulants use was heart diseases. One should realize that especially in such a group the anticoagulant therapy needs to be administered not only carefully but also individually.

Bladder cancer was the most common oncological problem diagnosed in the presented study. Carcinoma, urolithiasis, benign prostatic hyperplasia (BPH), and inflammatory or infectious etiologies are most commonly identified [6–10]. Even though one should think of bladder cancer firstly in case of hematuria, the symptom itself is present predominantly in patients on anticoagulants. Lower tract bleeding occurs in 57% of cases, with the majority of these being gross hematuria, while upper tract bleeding occurs in 40% of the cases, with twice as much gross as microscopic hematuria [11]. Previously published studies demonstrated that gross and microscopic hematuria in late 1960s were documented in 4 to 24% and 40%, respectively, of the patients on anticoagulant therapy with warfarin or heparin [12–14]. It was consistent with further studies from the 1990s, which revealed that gross hematuria occurred in 2% to 24% of patients receiving chronic anticoagulation with warfarin and/or aspirin for various indications [11, 15, 16]. Furthermore, it was aspirin that was in the majority of cases the reason of iatrogenic hematuria (78% versus 62% in the warfarin group) [16]. Several randomized controlled trials, which enrolled 15406 patients receiving heparins, revealed that the complication of hematuria was present in 1,6% of cases [17–23]. In the group treated with a high dose of LMW heparin, 5,8% of patients developed hematuria, while in the group treated with a low dose of LMW heparin, 0,4%. Furthermore, 4,7% of patients receiving a high dose of LDU heparin presented with hematuria versus 0,2% of patients receiving a low dose of LDU heparin. The incidence of hematuria in patients treated with fibrinolytic agents approached 20 to 30% [24]. The overall percentage of individuals on anticoagulants in the consulted patients was higher in our material due to the several reasons. First of all, the patients were enrolled in our study from the departments, in which admitted patients are in great need of such a therapy. Furthermore, the studies published so far are often based on the material from general population lacking urological point of view focused on the possibility of a tumor presence in urinary tracts.

Urologists are often asked to evaluate the need of diagnostics in patients with hematuria, who are on anticoagulant therapy. The commonly accepted diagnostic standard is based on clinical assessment, ultrasonography and/or excretory urography (IVP), and cystoscopy. During imaging and urological procedures, an etiology is found in 17 to 82% of cases [6–10]. In our study, only 19% of consultations proved to be of urological matter, and that fact supports strongly the opinion according to which the probability of finding a tumor in the group of patients on anticoagulants is very low. Approximately 2% to 5% of patients with microscopic hematuria and 10% of patients with gross hematuria have urothelial carcinoma [25, 26]. In a study of 1340 healthy men screened for hematuria, there was no correlation between the quantity of hematuria and urological disease severity [27]. Furthermore, hematuria produced by cancer and other serious diseases was frequently intermittent and appeared in small number of cases. In addition to that, the statistics change drastically along with enlarging the group of patients enrolled in the study and focusing on the conditions that individuals were treated from. In the large study of 1930 patients, nondiagnostic hematuria was found in the following: microscopic—68% and macroscopic hematuria—52%, respectively [28]. As mentioned above, these are arterial diseases, which due to the great need of anticoagulant therapy for patients suffering from those conditions and high prevalence of them in the population, coexist with iatrogenic hematuria [29]. Last and foremost is the fact that statistics in urological studies focus mainly on individuals referred by other clinicians to urological departments due to the highly possible urological origins of the patient's symptoms. In our study, we had a chance to examine the problem closely, due to the fact that the patients, who were consulted, suffered from different primary diseases.

Whether the prognosis with anticoagulants-associated hematuria is improved due to earlier detection of asymptomatic genitourinary lesions is uncertain [11]. Usually, the degree of hematuria is related to the degree of anticoagulation [11], although it may be the only manifestation of significant uropathological condition. Thus, some authors recommended a complete urological evaluation for all patients with nontraumatic anticoagulant-associated hematuria, having emphasized that a malignancy was found in 30% of the patients on anticoagulant therapy [11]. The statement that anticoagulants may serve as a potentiative enhancement for the detection of urological disease [11] seems to be quite risky nowadays, however. Moreover, as it was stated above, in our study, abnormalities of urinary tracts were found less frequently in patients receiving anticoagulants. The economic burden of investigating hematuria provokes a less intensive algorithm without loss of diagnostic efficacy [28]. However, some authors claimed that ultrasound in combination with IVP was recommended for maximal diagnostic efficacy [28]. In addition to that, cystoscopy shoud not be omitted on the basis of type of hematuria, age, or sex [28]. The American Urological Association recommended cystoscopy for all adults over 40 years old with microscopic hematuria and for those younger than 40 years with risk factors for developing bladder cancer [30]. However, such a policy leads to invasive procedures, for example, cystoscopy and imaging for 95% of patients with microscopic hematuria without malignancy detected [31]. The 3 types of hemorrhage that may occur in and around the urinary tract are retroperitoneal, intraluminal, and intrarenal [32]. Intraluminal bleeding often results in the formation of clots with possible painful passage or retention in the renal pelvis, which may simulate a neoplasm [33]. On the other hand, urothelial carcinoma of the bladder is frequently diagnosed in patients presenting with new-onset hematuria. The authors are of the opinion that if the hematuria is rarely the revelator of a tumor in patients on anticoagulant therapy, a suggestion appears to limit the standard diagnostics procedures in those patients. The new standard would comprise ultrasound examination of urinary tracts and cystoscopy to rule out renal and bladder cancer, while common DRE would be in favor of excluding BPH and advanced prostate cancer. The main stress, however, should be put on the cost and effectiveness analysis that was performed in our study. The high costs of a single neoplasm detection in patients presenting with hematuria are caused by the great number of cases of iatrogenic hematuria that have to be excluded during diagnostic procedures. The alarming results make it clear that guidelines for anticoagulant treatment should be reconsidered.

The other element that requires consideration is the relevance of the extent of anticoagulation, which is influenced by the indication for treatment and the patient's compliance, dietary status, and concomitant drug therapy [16]. Some authors claimed that the incidence of bleeding episodes is directly correlated to the PT [3, 7, 34]. Moreover, the incidence of gross hematuria correlates with the degree of anticoagulation [16]. In those studies, it was again emphasized that despite the undeniable effect of anticoagulants on iatrogenic hematuria occurrence, each case of hematuria deserved a full attention of a urologist. Authors claim, however, that the only way to reduce the number of iatrogenic hematuria, which is diagnostic matter at urological wards, is to address that message to the clinicians of other fields, who prescribe anticoagulants for their patients.

## 5. Conclusions

In our study, the strong correlation between the presence of hematuria and anticoagulant treatment was observed. Urological origins of hematuria are more often present in patients not receiving anticoagulant drugs. Standard urological diagnostic procedure, as an expensive and invasive action, should be engaged only after critical analysis of influence of anticoagulant drugs on the presence of hematuria. Authors suggest to redefine the present and future role of hematuria from a standard manifestation of serious urological disease to a common result of a long-term or high-dose anticoagulant therapy. The controversy that remains is how to perform differential diagnosis between “tumor-induced” hematuria and “postmedication” hematuria. Further studies on this topic would be beneficial for clinicians and health care providers.

## Figures and Tables

**Table 1:** Costs per patient according to National Health Fund.

Payer	USG of urinary tracts	CT of abdominal cavity and pelvis	Cytoscopy	Urography
National Heath Fund (NFZ)	9,3 EUR	66,67 EUR	158,67 EUR	52,4 EUR
* Overall (3-day hospitalization)*				*287 EUR*
